# Fe, Zn and Se Bioavailability in Chicken Meat Emulsions Enriched with Minerals, Hydroxytyrosol and Extra Virgin Olive Oil as Measured by Caco-2 Cell Model

**DOI:** 10.3390/nu10080969

**Published:** 2018-07-26

**Authors:** Lorena Martínez, Gaspar Ros, Gema Nieto

**Affiliations:** Department of Food Technology, Nutrition and Food Science, Veterinary Faculty University of Murcia, Campus de Espinardo, 30100 Murcia, Spain; lorena.martinez23@um.es (L.M.); gros@um.es (G.R.)

**Keywords:** Caco-2 cell model, Fe, Zn, Se, organic minerals, bioavailability, hydroxytyrosol

## Abstract

There is a high demand for functional meat products due to increasing concern about food and health. In this work, Zn and Se bioavailability was increased in chicken meat emulsions that are enriched with Hydroxytyrosol (HXT), a phenolic compound obtained from olive leaf. Six different chicken emulsions were elaborated. Three were made with broiler chicken meat supplemented with inorganic Zn and Se: control, one with HXT (50 ppm) added and one with HXT (50 ppm) and Extra Virgin Olive Oil (EVOO) (9.5%) added; and, three were made with chicken meat from chickens fed a diet that was supplemented with organic Zn and Se: control, one with HXT (50 ppm) added and one with HXT (50 ppm) and EVOO (9.5%) added. The samples were digested in vitro and the percent decomposition of phenolic compounds was measured by HPLC. Mineral availability (Fe, Zn and Se) was measured by cell culture of the Caco-2 cell line and the results were compared with mineral standards (Fe, Zn, and Se). The data obtained showed that neither HXT resistance to digestion nor Fe availability was affected by the presence of organic Zn and Se or phenolic compounds. Zn uptake increased in the presence of HXT, but not when its organic form was used, while Se uptake increased but it was not affected by the presence of HXT. It was concluded that the enrichment of meat—endogenously with organic minerals and exogenously with phenolic compounds—could be considered an interesting strategy for future research and applications in the current meat industry.

## 1. Introduction

Meat products are one of the most consumed food groups, representing 23% of the total Spanish meat market [1], while the international consumption of meat and meat products is gradually increasing, according to the last OECD/FAO (Organization for Economic Co-operation and Development/Food and Agriculture Organization of the United Nations) overview [2]. However, such habits are increasingly regarded as a problem since the International Agency for Research on Cancer (IARC) under the auspices of the World Health Organization (WHO) has classified processed meat as a carcinogen (Group I) and red meat as possible carcinogen (Group 2A) [3].

One way for improving the nutritional quality of meat and meat products, beef, lamb, pork, poultry, or chicken, among others, is to enrich them endogenously, through the feed that is provided to the animals. Micronutrient deficits represent a high sanitary cost for governments, for which reason, the last twenty years have seen an increase in research into food and soil fortification in an attempt to palliate these deficits. In the case of meat, Delles et al. [4] verified that the endogenous enrichment of chicken meat with Zn, Se, and vitamin E decreased lipid and protein oxidation, making it a good strategy for reducing the concentration of synthetic additives in meat and meat products. Moreover, mineral supplementation in broilers increases their performance, antioxidant enzyme activities, and the bioavailability of minerals, which also improves the nutritional quality of the meat [5,6].

The bioavailability of Fe, Zn, and Se minerals in some kinds of food has been studied both in vitro [7,8] and in vivo [9,10] with good results obtained in lamb, beef and chicken meat. As result, these important minerals for the immune system and the body function are more available in meat than in other kind of products [9].

On the other hand, synthetic additives, such as sulphites, BHT (butyl-hydroxytoluene), and BHA (butyl-hydroxyanisole), are usually added to meat product formulation in order to preserve them. Their use has increased social concern about food and health, based on research that correlates their consumption with the development of diseases and disorders (e.g., asthma, hyperactivity, cancer, etc.) [11,12,13]. The use of natural preservatives to increase the shelf life of meat is a promising tool because many plants (olive leaf, acerola, grape seed, cocoa, *Ginkgo biloba*, etc.) and spices (rosemary, green tea, black pepper, garlic, etc.) show antioxidant properties in meat products [14].

It is possible to provide meat product formulations with a healthier lipid profile through the substitution of animal fat by vegetable or fish oils, developing products with a lower content of saturated fatty acids (SFA) and LDL (low density lipoprotein)-cholesterol, while increasing the levels of unsaturated fatty acids (MUFA and PUFA) and improving the ω-6/ω-3 ratio. Extra Virgin Olive Oil (EVOO) can be used as substitute for animal fat due to its high content of unsaturated fatty acids (ω-9) and phenolic compounds, which prevent oxidative damage to the organism and reduce the risk of suffering diseases, such as cardiovascular disease, some cancers, Alzheimer’s, cataracts, among others [15].

However, when unsaturated fatty acids are added to meat products, they may produce greater oxidation that can be reduced with natural extracts. Hydroxytyrosol (HXT) is a phenethyloid with demonstrated in vitro antioxidant properties. It is commonly found in olive leaf and oil from this fruit and it is responsible for the intense flavour [16,17]. This extract has previously showed its antioxidant capacity in meat products that are rich in unsaturated fatty acids, like sausages and frankfurters containing HXT, nuts, and EVOO. Moreover, HXT binds to certain minerals, such as gluconate Fe (II) in black olives, which catalyzes the oxidation of this compound, so it is possible that HXT will affect the biological bioavailability of some minerals and trace elements [17].

For all of the above, the incorporation of minerals in animal feed and the use of natural antioxidants during the production of meat products can be considered to be a good strategy to replace synthetic additives.

The overall aim of this work was to test the nutritional and technological functionality of chicken emulsions made with chicken meat from broilers fed with Zn and Se (in both organic and inorganic forms), and subsequently enriched during processing with Extra Virgin Olive Oil (EVOO) and hydroxytyrosol (HXT). Functionality was evaluated by measuring HXT decomposition and the bioavailability of trace elements, such as Fe, Zn, or Se (absorption, transport, and metabolism) after in vitro digestion using a Caco-2 cell model.

## 2. Materials and Methods

### 2.1. Materials and Reagents

Reagents were prepared with deionized distilled water (Millipore Ibérica S.A., Barcelona, Spain). All of the materials used (bottles, tubes, spoons, etc.) were immersed in concentrated nitric acid for 24 h and washed three times with distilled deionized water before use, to avoid affecting the results due the presence of traces of minerals.

Enzymes and bile salts were purchased from Sigma Chemical Co. (St. Louis, MO, USA): Pepsin (Porcine: cat. No. P-7000), Pancreatin (Porcine: cat. no. B-8756), and bile salt (Porcine: cat. no. P-1750). Cell culture media, antibiotics (penicillin and streptomycin), glucose, 4-(2-hydroxyethyl)-1-piperazineethanosulfonic acid (HEPES), 2-(*N*-morpholino) ethanesulfonic acid (MES), and Hank’s Balanced Salt Solution (HBSS) were obtained from Gibco Life Technologies (Paisley, Scotland).

#### 2.1.1. Plant Extract

HXT extract of 23% purity was supplied by Nutrafur—Frutarom Group (Alcantarilla, Murcia, Spain) obtained from the vegetation water of olive trees. This extract is obtained by separating the oil left after wet centrifugation during fruit processing, while using a solvent extraction and purification process, including crystallization and clarification steps. The composition of this extract measured by HPLC is shown in Table 1.

#### 2.1.2. Animals and Diet

Because of the shorter average life of chickens, 40 days, animals were chosen to carry out this study. In addition, chicken meat was also chosen as a model due to it is high consumption around the world.

The following regulations were taken into account: EU regulation [18,19,20] and Spanish law 32/2007 [21] regarding the care of the animals, transport, experimentation, and slaughter for experimentation. The procedures that were used in the study were approved by the Bioethics Committee of Murcia University (authorization number: CEEA-92/2014). Three independent feeding trials (*n* = 3) over a one year period were performed. In each trial, 500 male broilers from 1 to 40 days old were randomly placed in two different floor once for each dietary group with 12 replicate pens for each diet. Each pen was assigned to two dietary treatments: basal diet—supplemented with inorganic Zn and Se (C) and basal diet supplemented with organic Zn and Se (SZ). From every independent trial, 12 replicate pens for each diet (2 animals were analysed per each pens and each diet). In total 24 (12 × 2) animal per diet (24 animals supplemented with inorganic Zn and Se and 24 animals with a basal diet supplemented with organic Zn and Se × 3 individual trial = 72 animals). The reference used for calculation of samples was reported by Festing & Altman [22]. The birds consumed feed and water at libitum. A starter diet containing 22% soybean meal and 3025 kcal/kg was provided up to 21 days of age and a grower diet from 21 to 42 days of age containing 21% soybean meal and 3105 kcal/kg. Both of the diets consisted of corn, wheat, whole soybeans, sunflower meal (28%), soybean oil, amino acids (methionine, threonine, and lysine), and minerals (CaHPO_4_, NaHCO_3_, and CaCO_3_). The control feed (C) was supplemented with inorganic minerals (0.3 ppm of Na_2_SeO_3_ and 80 ppm of ZnO), while the SZ feed was supplemented with organic minerals (0.2 ppm and 50 ppm of Se and Zn proteinate, respectively). After 40 days of feeding, two broilers from each pen were slaughtered. Both sides of the thighs were removed and skinned and then kept frozen for one week until the meat emulsion was made.

### 2.2. Samples

Two batches of chicken meat were used (from animals fed an organic or inorganic mineral diet) to elaborate meat emulsions, whose formulation incorporated HXT and EVOO, according to Table 2. Six different chicken emulsions were elaborated. Three were made with chicken meat from broilers that were fed a diet supplemented with inorganic Zn and Se: control (C), 50 ppm HXT (C_HXT_) and 50 ppm HXT and EVOO (9.5%) (C_HXTOl_); and, three were made with chicken meat supplemented with organic Zn and Se: control (SZ), 50 ppm HXT (SZ_HXT_) and 50 ppm HXT and EVOO (9.5%) (SZ_HXTOl_).

After mixing all of the ingredients, trimmed chicken meat was placed in a cutter and homogenised for 1 min or until a final temperature of 15 °C in a room at 4 °C (knife and bowl speeds 3000 and 10 rpm, respectively). Then, samples were cooked in a bath to reach an internal temperature of 75 °C. After cooking, they were left to cool at 4 °C.

### 2.3. In Vitro Digestion: Solubility Method

The digestion procedure was that described by Kanner & Lapidot [23], which is divided into two phases: gastric and intestinal digestion and performed at 37 °C. Samples were filtered (0.2 μm) and transferred to 50 mL polycarbonate ultracentrifugation tubes, where they were subjected to ultracentrifugation at 223,487 *g* at 4 °C for 95 min (L-100XP optimal Ultracentrifuge Beckman Coulter, rotor 70Ti). Finally, samples were kept at −80 °C until addition to the Caco-2 cell monolayer. Three replicate digestions were performed for each experiment.

The soluble fraction, both standards, and digested samples were preconditioned before addition to cell monolayer. HEPES and glucose were added at a final concentration of 5 mM and 50 mM, respectively, the pH was adjusted to 7.2–7.4 with 30% HCl and osmolarity was corrected with milli-Q water to 310 ± 10 mOsm kg^−1^ using a Vapor Pressure Osmometer (Wescor Vapro 5520).

### 2.4. Cell Culture Method

The Caco-2 cell line was from the European Collection of Cell Cultures (ECACC, number 86,010,202, Salisbury, UK). Tests were performed between passages 26 and 31. The culture medium was EMEM (Eagle Minimum Essential Medium) with phenol red, to which 10% fetal bovine serum, 1% glutamine, 1% nonessential amino acids, and 1% antibiotic (penicillin and streptomycin) were added. The cell line was maintained in 7.5 cm^2^ flasks, changing the medium every two days, and their growth was observed by inverted phase contrast microscopy. The flasks were kept at 37 °C with 5% CO_2_ at 95% humidity. A subculture was performed when the cell culture reached 80% confluence.

The cell monolayer viability was measured with an MTT assay [24] and a mycoplasma test to verify the purity of the cell line was performed. On the other hand, the cell monolayer integrity was evaluated by measuring the transepithelial electrical resistance (TEER) while using an apparent permeability test (phenol red test) [25].

### 2.5. Analytical Determinations: Study of Absorption, Transport and Metabolism in Caco-2 Cells

Cells were seeded on “Transwell” permeable polycarbonate supports with a 0.4 μm pore diameter and surface area of 3.8 cm^2^ in six-well plates. Cells were seeded at a density of 30,000 cells/cm^2^ changing the culture medium on alternate days. On the 13th day of subculture, the digested and preconditioned standards were added, in order to determine the absorption, transport, and mineral uptake by the cell monolayer during 1 h of incubation.

#### 2.5.1. Mineral Patterns

A catchment buffer composed of 0.75% NaCl, 0.074% KCl, 0.09% Glucose, 1.3% HEPES and 0.012% MgSO_4_ in milli-Q water adjusted to pH 7.2 was used as blank. Stock solutions of minerals were analyzed: 200 mM FeCl_3_·6H_2_O (nitrilotriacetic acid with 400 mM and 20 mM HCl) for Fe, 50 mM ZnSO_4_·7H_2_O for Zn, and 5 mM Na_2_SeO_3_ for Se. From these solutions, calibration curves were prepared with the catchment buffer.

#### 2.5.2. HXT Determination

The concentration of the phenolic compounds from the HXT extract was measured in the soluble fraction added to the cell monolayer, using a high performance liquid chromatograph (HPLC), following the method that was proposed by Benavente et al. [26]. When the amount of HXT from the soluble fraction was analysed, the decomposition percentage was calculated with respect to the total value added to the meat products.

The extract was dissolved in dimethylsulfoxide (DMSO) (5 mg/mL) and filtered through a nylon membrane of 0.45 μm diameter. Hewlett-Packard HP 1100 series equipped with a diode array detector was used. The stationary phase was a C18 LiChrospher^®^ 100 analytical column (250 × 4 mm) with a particle size of 5 μm (Merck, Darmstadt, Germany) that was heated to 30 °C. The flow rate was 1 mL/min and the absorption rate was monitored at 280 nm. The phenolic compounds of the extract were identified by a comparison of their retention times with the relevant standards and their UV spectrum was obtained by diode array.

#### 2.5.3. Fe, Zn and Se Determination

The mineral concentration of the samples was measured by plasma spectroscopy (ICP-OES) using the computer program ICAP THERMO DUO 6500. Inductively coupled plasma (ICP) is an ionized gas, electrically neutral, and confined in a discharge tube. Together with the optical emission spectrometer (OES), it forms the ICP-OES equipment. This is a technique for multi-element analysis using plasma source to dissociate atoms or ions of the sample, exciting them to a level at which they emit light (atomic emission spectroscopy) at a fixed wavelength. The spectra were dispersed by the diffraction grating, a detector measured the intensity of the emitted light and the concentration of minerals was calculated.

#### 2.5.4. Determination of Mineral Bioavailability in Caco-2 Cells

The differences between the mineral concentrations of the cell monolayer that was incubated with the standard solution of each mineral concentration and an unexposed monolayer (blank) provided an estimate of mineral retention. Transport percent was calculated as the difference between the amount of mineral in the basolateral chamber and the buffer that was used as blank. Mineral uptake was calculated by adding the retained and transported mineral. The percent of mineral retained and transported was calculated as the difference between mineral uptake and transport [27].

The equations used to calculate availability were:

% Retention = [retained compound (mg 100 g^−1^)/total compound (mg 100 g^−1^)] × 100

% Transport = [transported compound (mg 100 g^−1^)/total compound (mg 100 g^−1^)] × 100

% Uptake = [captured compound (mg 100 g^−1^)/total compound (mg 100 g^−1^)] × 100

Transport efficiency (TE) = [(% solubility) × (% transport)]/100

Uptake efficiency (UE) = [(% solubility) × (% uptake)]/100

### 2.6. Statistical Analysis

A descriptive analysis was performed and the results are expressed as AVG ± SD. The treatment effects and nutrient bioavailability were determined by analysis of variance (ANOVA) using pairs of factors. The homogeneity test that was used was the Scheefe test. The Statistix 8 programme was used to analyse the data considering as significant *p* < 0.05. The test applied to verify the normality of distribution was Shapiro Wilk and the results have been presented by mean values and SD.

## 3. Results and Discussion

### 3.1. Cell Culture

The cell line was checked by a mycoplasma test to ensure that it was free of contamination, which would have affected the results. The phenol red test was used to check the monolayer permeability. This confirmed the integrity of the cell membrane for mineral absorption experiments to be carried out (between days 8 and 21 of subculture). The data obtained were correlated directly with TEER (transepithelial electrical resistance) when the values were over 1000 Ω cm^2^, indicating that the monolayer was full [28]. The results of the MTT assay with different extracts added to the cell monolayer showed that the percentage of viability did not fall by more than 10%, so these solutions were not toxic to the cell line. The same conditions were applied in all of the experiments.

### 3.2. Mineral Content

Table 3 shows the mineral concentrations with normal distribution (M ± SD) measured in the broiler meat. The concentration of Zn and Se in SZ was significantly higher (20% and 88%, respectively) (*p* < 0.05) than in C, but no significant differences were found in the Fe content.

Similar results were obtained by Yan et al. [29] in meat from broiler chickens that were fed a diet supplemented with Zn. On the other hand, Wang & Xu [30] found no significant differences when supplementing diet of broiler chickens with organic Se.

### 3.3. HXT Degradation in an in Vitro Digester

Table 4 shows the concentration of HXT with normal distribution (M ± SD) in digested meat emulsions (soluble fraction added to Caco-2 cells), as measured by HPLC. Although HXT decomposition after digestion was very low, there were significant differences between samples (*p* < 0.05). For example, HXT degradation was 9.14% in C_HXT_ and 14.86% in SZ_HXT_, both higher than in samples C_HXTOl_ and SZ_HXTOl_, where losses of 3 and 1.04%, respectively, were recorded. This demonstrates that the total hydroxytyrosol content of this extract does not decrease when it is consumed in food products, as previously mentioned [31]. The results also show that HXT becomes more available when it is combined with EVOO, which is not surprising because both of the compounds share a common origin: the olive tree.

Similar results were obtained in other studies, which showed that the combination of HXT and EVOO maintained the antioxidant activity of phenolic compounds during cooking [32]. Similarly, Rubio et al. [33] demonstrated that HXT bioavailability in Caco-2/HepG2 cells was enhanced when it was combined with other extracts that are rich in phenolic compounds, such as thyme. 

However, in the SZ samples, the decomposition degree was greater than in C, in which HXT was not combined with EVOO. This could be due to interference between the organic and phenolic compounds from the HXT extract with Zn and Se. However, no information regarding this possible effect is available to compare the results of this study. The affinity of HXT for certain minerals has been reported previously. For example, Ca absorption increases with HXT and EVOO consumption in osteoporosis patients, preventing the bone loss [31]. On the other hand, HXT is bound to Fe (II) in black olives [17], so this compound can be associated with another mineral forms, such as Zn or Se.

### 3.4. Fe Bioaccessibility

To assess Fe bioaccessibility, Table 5 shows the results of Fe retention, transport, and uptake with normal distribution (M ± SD) in Caco-2 cells after adding the soluble fraction from chicken emulsions. There were no significant differences between the Fe absorption values in the samples. However, there were significant differences in basal Fe concentrations and the retained and transported mineral between the different samples. Moreover, C and SZ had a higher percent of mineral uptake (*p* < 0.05), while C_HXT_, C_HXTOl_, SZ_HXT_ and SZ_HXTOl_ showed higher percentages of mineral transport (*p* < 0.05). In the same way, the transport and uptake efficiencies behaved similarly, being higher than 7.5% in C_HXT_ and SZ_HXT_ and more than 12% in C_HXTOl_ and SZ_HXTOl_. This may be because HXT increased Fe transport from the apical to the basolateral chamber. This may be due to the great affinity of HXT bind to Fe, as was observed by Wang et al. [17]. In this research, it was observed how HXT binds with gluconate Fe (II) in black olives, which catalyses the oxidation of this mineral. Moreover, similar results concerning Fe availability were obtained by Soresen & Bukhave [34] and Pachón et al. [35] with enriched pork and chicken meat, respectively, in Caco-2 cells.

### 3.5. Zn Bioaccessibility

Table 6 shows the results that were obtained for Zn retention, transport, and uptake with normal distribution (M ± SD) in Caco-2 cells after adding the soluble fraction to the cell monolayer. 

As expected, the SZ samples showed a higher basal concentration of this mineral than C samples (*p* < 0.05). On the other hand, C_HXT_, C_HXTOl_, SZ_HXT_, and SZ_HXTOl_ showed greater Zn bioavailability than C and SZ (*p* < 0.05). So, the mineral retention percent in the apical chamber was higher in C and SZ samples, reaching 51% in the SZ batch. However, the mineral uptake by Caco-2 cells was significantly lower in C_HXT_ and SZ_HXT_, reaching 17% and 25 to 30% in C and SZ, respectively. For its part, the transported mineral percent was constant in the SZ (about 30%) and higher in C, where it was more variable (24–37%).

These results disagree to some extent with those of other researchers, such as, Frontela et al. [27] or Frontela et al. [36], who observed an increase in Zn absorption in milk formulas enriched with Fe, Zn and Ca. No information exists in the literature concerning the bioavailability of Zn in Caco-*2* using enriched meat, making it an interesting topic for further research.

In addition, the RDA of Zn for a healthy adult is among 8 and 12 mg/day [36], which according to the quantity ingested, the consumption of 100 g of SZ_HXTOl_ supposes 5% of this RDA, while 100 g of C_HXTOl_ supposes 2%. So, it can be concluded that consumption of this kind of products helps to reach the recommendation, but it is necessary complete the diet with other products that are rich in Zn, such as oat, mussels, or cockles.

### 3.6. Se Bioaccessibility

Table 7 shows the results of Se retention, transport, and uptake with normal distribution (M ± SD) in Caco-2 cells after adding digested chicken emulsions. The table also shows an estimate of its availability and the values of mineral transport and uptake efficiency.

In this case, no significant differences were found when HXT or EVOO were incorporated in the formulas (C_HXT_, C_HXTOl_, SZ_HXT_, and SZ_HXTOl_). However, there was a slight increase (*p* < 0.05) in the Se initial concentration of Se (0.01 mg/mL higher) and Se uptake (8.31% higher) by Caco-2 cells in SZ samples made with chicken meat enriched with organic Se.

These results are similar to others concerning Se bioavailability in seafoods in Caco-2 cells [37,38]. The bioavailability of Se in the intestine is very low, and its absorption efficiency does not exceed 10%. Although no information on Se bioavailability in enriched meats has been found, the results that were obtained suggest that the food matrix used is not a dependent factor for its availability, because mineral uptake is also low in fish and seafoods. In addition, HXT is not an influential factor, because of the retention, transport, and uptake values are not affected by its presence, in the same way as Zn bioavailability. This observation can be explained by previous research that has shown how HXT increases Fe and Ca bioavailability [17,31]. So, if HXT acts as transporter of Ca and Fe, which are competitors of Zn and Se, it can be concluded that Fe acts as a competitor for binding with HXT, preventing the absorption of Zn and Se bound with this phenolic compound. Consequently, as can be appreciated, Fe availability in this study was higher in the samples with HXT, while the uptake of Zn and Se combined with HXT was lower.

In addition, the RDA of Se for a healthy adult is among 55 and 70 µg/day [37], that according to the quantity ingested, the consumption of 100 g of SZ samples supposes the 100% of this RDA. So, it can be concluded that consumption of this kind of products helps to reach the recommendation about this essential mineral.

Although this research was carefully prepared, this study presents some limitations. One of the main limitations is the scarce number of animals (*n* = 70). Another limitation derived from cell culture methods. These vary in their reproducibility and characterization. On the other hand, the cell culture model is compared with the digestive system of the human body, which is more complex and may vary significantly. However, through an exhaustive bibliographical research, the relationships that occurred during the absorption process of minerals and phenolic compounds have been justified.

## 4. Conclusions

When comparing the results that were obtained for the different tissues, the SZ samples, enriched with organic forms of Zn and Se, showed higher levels of bioavailability of these trace elements than the C samples, made from the meat of chickens fed inorganic forms of the same minerals (except in the case of Fe). This would confirm that organic forms of Zn and Se are more bioavailable in meat, as has been demonstrated in other kinds of matrix. However, it has also been showed how the presence of HXT, a phenolic compound, helps to increase Fe absorption by binding to it, while it prevents Zn and Se absorption in foods with a high Fe content, such as meat. In conclusion, the enrichment of meat endogenously with organic minerals or exogenously with phenolic compounds may be considered an interesting strategy for future applications in the meat industry: either for making meat products with a high content of minerals (Fe, Zn, and Se) or meat products with a high content of Fe and rich in HXT with its potential antioxidant power.

## Figures and Tables

**Table 1:** Hydroxytyrosol (HXT) extract composition measured by high performance liquid chromatograph (HPLC).

Sample	HXT	
Compounds	Retention Time (min)	% Absolute
Hydroxytyrosol	5.68	82.79
Tyrosol	8.5	9.36
Dimer of Hydroxytyrosol	13.31	5.35
Verbascoside	17.8	2.5

HXT: extract of Hydroxytyrosol 23% vegetation waste water.

**Table 2:** Ingredients (g) of chicken emulsion samples.

Ingredients (g)	Chicken Meat					
	Enriched with Inorganic Forms of Zn and Se			Enriched with Organic Forms of Zn and Se		
	**C**	**C_HXT_**	**C_HXTOl_**	**SZ**	**SZ_HXT_**	**SZ_HXTOl_**
Chicken meat (g)	713	713	616	713	713	616
HXT (ppm)	0	50	50	0	50	50
EVOO (mL) ^1^	0	0	100	0	0	100
Water (mL)	172	172	172	172	172	172
Ice (g)	100	100	100	100	100	100
Salt (g NaCl)	15	15	15	15	15	15
**Total**	**1000**	**1050**	**1053**	**1000**	**1050**	**1053**

HXT: Hydroxytyrosol (23% extract from vegetation waste water). EVOO: Extra Virgin Olive Oil. C: Control; C_HXT_: 50 ppm HTX; C_HXTOl_: 50 ppm HXT + 10% EVOO; SZ: Control fortified with Zn and Se meat; SZ_HXT_: SZ + 50 ppm HXT; SZ_HXTOl_: SZ + 50 ppm HXT + 10% EVOO. ^1^ Emulsion made with olive oil and 3% soy lecithin (see materials and methods section for further details).

**Table 3:** Initial mineral concentration in meat.

Sample	Experimental Treatments	
	C (M ± SD)	SZ (M ± SD)
Fe Mineral sample (mg/kg)	10.65 ± 0.04	10.32 ± 0.02
Zn Mineral sample (mg/kg)	1.491 ± 0.005 ^b^	1.788 ± 0.08 ^a^
Se Mineral sample (mg/kg)	0.149 ± 0.04 ^b^	0.281± 0.0 ^a^

C: Control meat; SZ: Control meat fortified with organic Zn and Se; M ± SD: Mean ± standard deviation. Different letters in the same row indicate significant differences between samples (*p* < 0.05).

**Table 4:** HXT concentration in emulsions (soluble fraction added to Caco-2 cells) measured by HPLC.

	Experimental Treatments					
	C (M ± SD)	C_HXT_ (M ± SD)	C_HXTOl_ (M ± SD)	SZ (M ± SD)	SZ_HXT_ (M ± SD)	SZ_HXTOl_ (M ± SD)
HXT added to meat (ppm)	0.0 ± 0.0 ^b^	50 ± 0.0 ^a^	50 ± 0.0 ^a^	0.0 ± 0.0 ^b^	50 ± 0.0 ^a^	50 ± 0.0 ^a^
Digested HXT concentration (ppm)	0.0 ± 0.0 ^e^	45.43 ± 0.01 ^c^	48.5 ± 0.01 ^b^	0.0 ± 0.0 ^e^	42.57 ± 0.02 ^d^	49.48 ± 0.01 ^a^
% Decomposition	0.0 ± 0.0 ^e^	9.14 ± 0.01 ^b^	3 ± 0.01 ^c^	0.0 ± 0.0 ^e^	14.86 ± 0.02 ^a^	1.04 ± 0.01 ^d^

M ± SD: Mean ± standard deviation; HXT: Hydroxytyrosol (23% extract from vegetation waste water). EVOO: Extra Virgin Olive Oil. C: Control; C_HXT_: 50 ppm HTX; C_HXTOl_: 50 ppm HXT + 10% EVOO; SZ: Control fortified with Zn and Se meat; SZ_HXT_: SZ + 50 ppm HXT; SZ_HXTOl_: SZ + 50 ppm HXT + 10% EVOO. Different letters in the same row indicate significant differences between samples (*p* < 0.05).

**Table 5:** Fe retention, transport, and cellular uptake in enriched emulsions.

	Experimental Treatments					
	C (M ± SD)	C_HXT_ (M ± SD)	C_HXTOl_ (M ± SD)	SZ (M ± SD)	SZ_HXT_ (M ± SD)	SZ_HXTOl_ (M ± SD)
Fe concentration in the soluble fraction added (mg/mL)	0.27 ± 0.01 ^d^	0.33 ± 0.01 ^c^	0.25 ± 0.01 ^d^	0.44 ± 0.01 ^b^	0.43 ± 0.01 ^b^	0.49 ± 0.01 ^a^
Mineral added monolayer (μg)	4.32 ± 0.01 ^d^	4.51 ± 0.02 ^c^	4.64 ± 0.07 ^b^	4.3 ± 0.001 ^d^	4.88 ± 0.0009 ^b^	4.95 ± 0.07 ^a^
Mineral retained in apical chamber (μg)	1.13 ± 0.08 ^b^	1.15 ± 0.03 ^ab^	1.18 ± 0.08 ^ab^	1.12 ± 0.0001 ^b^	1.21 ± 0.0008 ^a^	1.15 ± 0.08 ^ab^
Retention %	26.16 ± 0.4 ^a^	25.5 ± 0.3 ^b^	25.43 ± 0.35 ^b^	26.05 ± 0.55 ^a^	24.8 ± 0.37 ^c^	23.23 ± 0.58 ^c^
Mineral transported to basolateral chamber (μg)	1.06 ± 0.003 ^c^	1.32 ± 0.0 ^b^	1.37 ± 0.0 ^b^	1.17 ± 0.0001 ^c^	1.52 ± 0.09 ^a^	1.54 ± 0.03 ^a^
Transport %	24.54 ± 0.42 ^f^	29.27 ± 0.18 ^d^	29.52 ± 0.08 ^c^	27.21 ± 0.04 ^e^	31.15 ± 0.4 ^a^	31.11 ± 0.2 ^b^
Mineral uptake by cells (μg)	2.13 ± 0.0001 ^bc^	2.03 ± 0.0004 ^d^	2.09 ± 0.1 ^c^	2.01 ± 0.08 ^de^	2.15 ± 0.11 ^b^	2.26 ± 0.1 ^a^
Uptake %	49.31 ± 1.98 ^a^	45.01 ± 3.08 ^cd^	45.04 ± 4.39 ^cd^	46.74 ± 4.03 ^b^	44.05 ± 4.26 ^d^	45.65 ± 2.13 ^c^
TE	6.42 ± 1.91 ^d^	7.46 ± 0.11 ^bc^	7.5 ± 0.37 ^b^	7.08 ± 0.16 ^c^	7.72 ± 0.33 ^a^	7.22 ± 0.18 ^cd^
UE	12.89 ± 1.17 ^a^	11.48 ± 2.95 ^b^	11.45 ± 2.34 ^b^	12.17 ± 0.21 ^ab^	10.92 ± 2.05 ^c^	10.6 ± 1.13 ^c^

M ± SD: Mean ± standard deviation; TE: Transport efficiency; UE: Uptake efficiency. HXT: Hydroxytyrosol (23% extract from vegetation waste water). EVOO: Extra Virgin Olive Oil. C: Control; C_HXT_: 50 ppm HTX; C_HXTOl_: 50 ppm HXT + 10% EVOO; SZ: Control fortified with Zn and Se meat; SZ_HXT_: SZ + 50 ppm HXT; SZ_HXTOl_: SZ + 50 ppm HXT + 10% EVOO. Different letters in the same row indicate significant differences between samples (*p* < 0.05).

**Table 6:** Zn retention, transport, and cellular uptake in enriched emulsions.

	Experimental Treatments					
	C (M ± SD)	C_HXT_ (M ± SD)	C_HXTOl_ (M ± SD)	SZ (M ± SD)	SZ_HXT_ (M ± SD)	SZ_HXTOl_ (M ± SD)
Zn concentration in the soluble fraction added (mg/mL)	0.14 ± 0.01 ^e^	0.16 ± 0.01 ^de^	0.18 ± 0.01 ^cd^	0.21 ± 0.01 ^bc^	0.22 ± 0.006 ^b^	0.49 ± 0.01 ^a^
Mineral added monolayer (μg)	3.79 ± 0.02 ^e^	4.49 ± 0.11 ^c^	6.04 ± 0.27 ^b^	4.07 ± 0.1 ^d^	4.63 ± 0.06 ^c^	4.95 ± 0.07 ^a^
Mineral retained in apical chamber (μg)	1.7 ± 0.02 ^c^	2.23 ± 0.1 ^bc^	2.73 ± 0.26 ^ab^	1.8 ± 0.11 ^c^	2.4 ± 0.06 ^cd^	1.15 ± 0.08 ^ab^
Retention %	44.85 ± 0.18 ^d^	49.66 ± 0.01 ^b^	45.19 ± 0.16 ^c^	44.23 ± 0.2 ^d^	51.83 ± 0.61 ^a^	23.23 ± 0.58 ^c^
Mineral transported to basolateral chamber (μg)	0.94 ± 0.0001 ^c^	1.29 ± 0.12 ^bc^	2.23 ± 0.18 ^a^	1.23 ± 0.13 ^bc^	1.4 ± 0.12 ^b^	1.54 ± 0.03 ^a^
Transport %	24.8 ± 0.07 ^e^	28.73 ± 0.01 ^d^	36.92 ± 0.01 ^a^	30.22 ± 0.009 ^c^	30.23 ± 0.009 ^c^	31.11 ± 0.2 ^b^
Mineral uptake by cells (μg)	1.15 ± 0.1 ^ab^	0.97 ± 0.06 ^bc^	1.08 ± 0.06 ^ab^	1.04 ± 0.005 ^abc^	0.83 ± 0.0 ^c^	2.26 ± 0.1 ^a^
Uptake %	30.34 ± 4.17 ^a^	21.6 ± 1.32 ^c^	17.88 ± 3.9 ^d^	25.55 ± 1.26 ^b^	17.92 ± 0.94 ^d^	45.65 ± 2.13 ^c^
TE	11.12 ± 0.03 ^e^	14.26 ± 0.02 ^c^	16.68 ± 0.21 ^a^	13.36 ± 0.3 ^d^	15.66 ± 0.12 ^b^	7.22 ± 0.18 ^cd^
UE	13.6 ± 1.87 ^a^	10.72 ± 3.27 ^c^	8.07 ± 6.51 ^e^	11.3 ± 2.35 ^b^	9.28 ± 1.01 ^d^	10.6 ± 1.13 ^c^

M ± SD: Mean ± standard deviation; TE: Transport efficiency; UE: Uptake efficiency; HXT: Hydroxytyrosol (23% extract from vegetation waste water). EVOO: Extra Virgin Olive Oil; C: Control; C_HXT_: 50 ppm HTX; C_HXTOl_: 50 ppm HXT + 10% EVOO; SZ: Control fortified with Zn and Se meat; SZ_HXT_: SZ + 50 ppm HXT; SZ_HXTOl_: SZ + 50 ppm HXT + 10% EVOO. Different letters in the same row indicate significant differences between samples (*p* < 0.05).

**Table 7:** Se retention, transport, and cellular uptake in enriched emulsions.

	Experimental Treatments					
	C (M ± SD)	C_HXT_ (M ± SD)	C_HXTOl_ (M ± SD)	SZ (M ± SD)	SZ_HXT_ (M ± SD)	SZ_HXTOl_ (M ± SD)
Se concentration in the soluble fraction added (mg/mL)	0.01 ± 0.0 ^b^	0.01 ± 0.0 ^b^	0.01 ± 0.0 ^b^	0.02 ± 0.0001 ^a^	0.01 ± 0.0 ^b^	0.49 ± 0.01 ^a^
Mineral added monolayer (μg)	4.5 ± 0.0	4.5 ± 0.0	4.5 ± 0.0	4.5 ± 0.0	4.5 ± 0.0	4.95 ± 0.07 ^a^
Mineral retained in apical chamber (μg)	1.5 ± 0.0 ^a^	1.5 ± 0.0 ^a^	1.5 ± 0.0 ^a^	1.31 ± 0.0 ^b^	1.5 ± 0.0 ^a^	1.15 ± 0.08 ^ab^
Retention %	33.33 ± 0.0 ^a^	33.33 ± 0.0 ^a^	33.33 ± 0.0 ^a^	29.18 ± 0.98 ^b^	33.33 ± 0.0 ^a^	23.23 ± 0.58 ^c^
Mineral transported to basolateral chamber (μg)	1.5 ± 0.0 ^a^	1.5 ± 0.0 ^a^	1.5 ± 0.0 ^a^	1.31 ± 0.0 ^b^	1.5 ± 0.0 ^a^	1.54 ± 0.03 ^a^
Transport %	33.33 ± 0.0 ^a^	33.33 ± 0.0 ^a^	33.33 ± 0.0 ^a^	29.18 ± 0.01 ^b^	33.33 ± 0.0 ^a^	31.11 ± 0.2 ^b^
Mineral uptake by cells (μg)	1.5 ± 0.0 ^b^	1.5 ± 0.0 ^b^	1.5 ± 0.0 ^b^	1.87 ± 0.0 ^a^	1.5 ± 0.0 ^b^	2.26 ± 0.1 ^a^
Uptake %	33.33 ± 0.0 ^b^	33.33 ± 0.0 ^b^	33.33 ± 0.0 ^b^	41.64 ± 0.69 ^a^	33.33 ± 0.0 ^b^	45.65 ± 2.13 ^c^
TE	11.10 ± 0.0 ^a^	11.10 ± 0.0 ^a^	11.10 ± 0.0 ^a^	8.51 ± 0.1 ^b^	11.10 ± 0.0 ^a^	7.22 ± 0.18 ^cd^
UE	11.10 ± 0.0 ^b^	11.10 ± 0.0 ^b^	11.10 ± 0.0 ^b^	12.15 ± 0.19 ^a^	11.10 ± 0.0 ^b^	10.6 ± 1.13 ^c^

M ± SD: Mean ± standard deviation; TE: Transport efficiency; UE: Uptake efficiency. HXT: Hydroxytyrosol (23% extract from vegetation waste water). EVOO: Extra Virgin Olive Oil. C: Control; C_HXT_: 50 ppm HTX; C_HXTOl_: 50 ppm HXT + 10% EVOO; SZ: Control fortified with Zn and Se meat; SZ_HXT_: SZ + 50 ppm HXT; SZ_HXTOl_: SZ + 50 ppm HXT + 10% EVOO. Different letters in the same row indicate significant differences between samples (*p* < 0.05).
